# Examining student burnout causes among English as a foreign language students: focus on school climate and student growth mindset

**DOI:** 10.3389/fpsyg.2023.1166408

**Published:** 2023-05-12

**Authors:** Xiaoling Liu

**Affiliations:** Department of Foreign Language, Anyang Institute of Technology, Anyang, China

**Keywords:** student burnout, school climate, growth mindset, EFL, SEM

## Abstract

**Introduction:**

The aim of this study was to investigate the relationship between student burnout and two key factors - perceived school climate and growth mindset - in the context of English as a foreign language (EFL) learning among Chinese students.

**Methods:**

A sample of 412 intermediate English language learners from China participated in an online survey and completed valid measures of the three constructs. Confirmatory factor analysis (CFA) was used to establish the validity of the scales used to measure the three latent variables. Structural equation modeling (SEM) was then used to test the proposed model.

**Results:**

The results of SEM showed that both perceived school climate and growth mindset had a significant positive impact on EFL student burnout, with perceived school climate having a stronger effect compared to growth mindset.

**Discussion:**

The findings suggest that promoting a positive school climate and fostering a student growth mindset can help reduce student burnout in EFL settings.

## 1. Introduction

The prevalence of student burnout among students is a significant issue due to the numerous negative outcomes it can lead to, such as socioeconomic, relational, and socio-professional problems (Schaufeli et al., 2002; Maroco and Tecedeiro, 2009; Derakhshan et al., 2022a). According to Cornér et al. (2017), student burnout can occur as a result of increased workload and long-term academic pressure. The development of relevant capabilities and academic well-being is crucial for a high-performing educational process in today’s universities (Zhang and Zhang, 2020; Derakhshan et al., 2022a).

The significance of a healthy school climate, school supportiveness, and positive motivation from teachers has been acknowledged in education as it positively impacts students’ academic performance (Caldarella et al., 2011; Meylan et al., 2020; Grazia, 2022). Optimal learning is directly related to the level of support provided by the school environment for students’ emotional, social, esthetic, and psychomotor development (Cohen et al., 2009; Faour, 2012; Ozdemir, 2012). School climate, as defined by Cohen et al. (2009), encompasses the quality and experiences of school life, including intangible aspects such as relational emotions and emotional attachment, as well as tangible aspects such as daily interactions between teachers and students and educational practices (Reyes et al., 2012). It encompasses all internal aspects that distinguish between different types of schools and have a profound impact on behavior in schools (Hoy and Miskel, 2012). The promotion of a high-quality climate is influenced by factors such as teaching practices, achievement objectives, curriculum, and teacher development (Shindler et al., 2016).

The significance of second language (L2) learning in school has prompted numerous researchers to examine the impact of school climate on students’ academic achievement and motivation for learning. Malinen and Savolainen (2016) and Salmela-Aro et al. (2008) have explored the positive and negative aspects of school climate and their association with students’ academic outcomes. Positive aspects of school climate include factors such as interest, social and academic development, communication and participation opportunities, and unity. On the other hand, negative aspects of school climate can include confusion in communication, aggressive attitudes and negative behavior of teachers, resistance to change, neglect of student needs, and conventional decision-making. School climate can also shape students’ mindset and evolve as they progress through higher grades, with a focus on ability and performance (Eccles and Roeser, 2011; Wang and Degol, 2016). Dweck (2009) has identified two types of mindsets: fixed and growth. A fixed mindset is a belief that students’ traits (e.g., intelligence, talent, and ability) are unchanging, whereas a growth mindset is a belief that these traits can be developed and improved through effort and innate abilities (Dweck, 2006; Blackwell et al., 2007; Kim, 2020; Yu et al., 2022; Derakhshan et al., 2022b).

The outcomes of a fixed mindset can include weak intrinsic motivation, low self-efficacy, academic incompetence, and goal loss. Conversely, students with a growth mindset are more likely to pursue their long-term goals with confidence, leading to improved academic achievement, even in the face of academic challenges and failures (Dweck, 2006; Gross-Loh, 2015; McClendon et al., 2017; Yu et al., 2022). As a result, a growth mindset may help mitigate student burnout (Dweck, 2006; Zeng et al., 2016).

Although the significance of growth mindset and school climate in relation to student burnout has been established sporadically in the literature (e.g., Yeager and Dweck, 2012; Zeng et al., 2016; Fatou and Kubiszewski, 2018), the specific contributions of this study include the examination of the relationship between these two factors and student burnout in a Chinese EFL context. To our knowledge, no previous study has examined these factors simultaneously in this specific context. Moreover, our study aims to provide insights into the potential role of school climate and growth mindset in mitigating student burnout, thus contributing to the development of effective interventions and strategies to address this issue. By investigating the impact of school climate and growth mindset on student burnout, this study aims to enhance our understanding of the factors that may influence academic well-being, and to provide practical implications for educators and policymakers.

Furthermore, the choice to explore the relationship between these constructs specifically for EFL students warrants further attention. Although previous studies have examined these variables in various academic contexts, little is known about how they may manifest among EFL students. EFL students may face unique challenges related to language proficiency, cultural differences, and academic expectations (Li et al., 2021), which could impact their experiences with burnout, school climate, and growth mindset. Therefore, investigating these variables in the context of EFL students is crucial for understanding their potential impact on academic outcomes and overall well-being in this population.

## 2. Literature review

### 2.1. Burnout

In recent years, the phenomenon of burnout has transcended beyond professional contexts and has garnered widespread attention (Farina et al., 2020; Hyer et al., 2020; Bing et al., 2022). Burnout is a complex construct that can be described as a psychological syndrome characterized by long-term exhaustion and disinterest in one’s work or activities (Schaufeli et al., 2009). As per the findings of Han et al. (2020) and based on Maslach et al.’s (1997) model, burnout comprises three crucial dimensions – emotional exhaustion, depersonalization, and reduction in personal accomplishment. Emotional exhaustion encompasses feelings of emotional drain, overexertion, and fatigue as a result of an excessive workload (Han et al., 2020; Sato et al., 2022). Depersonalization involves a detachment from others’ emotions and developing unemotional tendencies in response to their distress (Allen et al., 2018). Furthermore, reduction in personal accomplishment refers to a lack of satisfaction and motivation in one’s achievements, resulting from a diminished sense of accomplishment (Jacobs and Dodd, 2003). Overall, as discussed by several scholars (e.g., Maslach et al., 2001; Schaufeli et al., 2002), the long-term effects of burnout can have serious implications on an individual’s mental well-being. In particular, the outcomes of burnout, such as the increased risk of depression, low self-esteem, and elevated risk of suicide, can have a significant impact on the academic performance and overall success of students (Eriksson et al., 2011; Mutkins et al., 2011; Li et al., 2021).

Burnout, as a result of chronic interpersonal strain, can be viewed as a manifestation of an individual’s subpar performance in the workplace and its subsequent detrimental effects on their physical and mental health, behavior, and attitudes (Maroco and Campos, 2012; Paloș et al., 2019; Salgado and Au-Yong-Oliveira, 2021). This is particularly evident in the teaching profession where a detached culture can lead to high levels of burnout and emotional exhaustion among teachers who struggle to cope with their anxieties in private (Küçükoğlu, 2014; Shen et al., 2015; Saloviita and Pakarinen, 2021; Wang, 2022). The phenomenon of teacher burnout is, therefore, shaped by the teacher’s personality characteristics (Pietarinen et al., 2013). As described by Blasé (1982), teacher burnout is defined as a result of “prolonged job strain that results from the inadequacy of coping resources and the absence of equitable rewards in relation to the demands of work-related stressors” (p. 109).

Chronic emotional stress, physical exhaustion, low motivation, low academic performance, and neglect toward clients are factors that contribute to burnout (Prada-Ospina, 2019). Among these, stress is a significant factor that negatively impacts academic performance. Consequently, students are more susceptible to academic burnout (Schaufeli et al., 2002). Academic burnout can be described as a response to stress in the university community that affects “the development, understanding, and satisfaction of the students with their education and academic life” (Dominguez and Breso, 2015, p. 4).

As highlighted by researchers such as Maroco and Tecedeiro (2009) and Merino-Soto and Fernández-Arata (2017), academic burnout among students is characterized by their perceptions of cognitive and emotional exhaustion as a result of demanding university obligations, such as class attendance, assignment completion, exam preparation, active participation in class, and interaction with peers and educational institutions. The excessive workload in academic contexts takes a toll on students’ physical and mental health and affects their academic success as they lack the mental structure to appropriately respond, leading to a lack of competency and self-efficacy, along with disillusionment toward teachers, peers, and educational pursuits (Maroco and Tecedeiro, 2009).

In this study, student burnout refers to a state of physical and emotional exhaustion resulting from academic demands and responsibilities. It is a multi-faceted concept that includes three key dimensions: exhaustion, cynicism, and inefficacy (Maslach et al., 2001). Students also experience burnout due to high levels of academic demands and responsibilities (Derakhshan et al., 2022a). In China, Hu and Schaufeli (2009) identified that students were likely to experience burnout in an exam-oriented teaching environment, which they categorized into three dimensions: exhaustion related to study demands, cynicism related to detachment from studies, and efficacy related to academic achievement.

### 2.2. School climate

The construct of school climate, particularly in the context of L2 learning, is a crucial area of interest as it has a significant impact on individuals’ well-being and academic achievement outcomes (Thapa et al., 2013). A positive school climate is particularly noteworthy as it serves as a protective factor for students with poor academic performance and those from lower socio-economic backgrounds (Thapa et al., 2013; Dutta and Sahney, 2016; Lombardi et al., 2019; Jia and Konold, 2021; Mohammad Hosseini et al., 2022). The dimensions of school climate, such as safety, educational practices, social relationships, school facilities, and school connectedness, significantly influence students and their educational paths (Thapa et al., 2013; Grazia and Molinari, 2020).

The aspect of safety encompasses feelings of security, adherence to rules and norms, care for others, positive learning environments, and healthy development (Goldstein et al., 2008; Capp et al., 2020). Educational practices encompass intellectual engagement, self-awareness, supportive and productive learning environments, positive changes in student attitudes and academic behavior, and social, emotional, and ethical learning (Gage et al., 2016). Social relationships refer to interactions with parents, school involvement and management, and collaborative and supportive connections with others (Bradshaw et al., 2014; Gage et al., 2014). School facilities include a physical layout of the school as well as accessible resources and supplies (Gislason, 2010). School connectedness refers to student support, belongingness to a positive peer group, commitment to educational objectives, positive educational outcomes, and positive health outcomes (García-Moya et al., 2019).

Overall, an unremitting positive school climate vigorously develops students’ cooperative learning and enhances their motivation, as well as their healthy relationships accompanied by psychological well-being, high self-esteem, and buoyancy (Thapa et al., 2013; Cornell and Huang, 2016). More specifically, a positive school climate can promote a high level of students’ engagement as well as academic achievement, thereby reducing levels of burnout among students (Berkowitz et al., 2017; Fan and Wang, 2022).

### 2.3. Growth mindset

The concept of incremental and entity theories was first introduced by Blackwell et al. (2007). The incremental theory suggests that individuals can grow and develop through challenges and obstacles, while the entity theory holds that individuals have a fixed level of ability and are limited by their innate characteristics. As a result of these differing views, two distinct psychological perspectives, the growth mindset, and the fixed mindset have been established (Dweck, 2012; Yeager and Dweck, 2012). The growth mindset has been found to promote resilience, while the fixed mindset is associated with stagnation (Dweck, 2012; Burnette et al., 2013; Dweck and Yeager, 2019; Zeng et al., 2019; Holzberger et al., 2021).

From a positive psychological perspective (Wang et al., 2021), the role of mindset in dealing with existential crises and promoting positive change has been emphasized (Wong, 2010; Waters et al., 2022). Researchers such as Dweck (2006) and Ronkainen et al. (2019) have found that an individual’s growth mindset is directly linked to their ability to recognize and overcome challenges, and their tendency to work hard. The academic performance of students has also been linked to their growth mindset, as students with a growth mindset are more likely to adopt new strategies and display greater creativity in their academic pursuits (Slavich and Zimbardo, 2012; Claro et al., 2016; Xu et al., 2021; Zhang et al., 2022). Additionally, students with a higher level of growth mindset are less susceptible to academic burnout (Kim, 2020). Teachers who adopt a growth mindset can also influence their students’ beliefs about their competence and capability, encouraging students to put in more effort and achieve their goals (Schmidt et al., 2015; Degol et al., 2018; Zeng et al., 2019; Zhang et al., 2022).

### 2.4. Empirical background

A number of studies have explored the phenomenon of burnout and have identified a set of constructs, such as learning engagement, school climate, growth mindset, language learning motivation, resilience, self-efficacy, and academic grit, as important in the evolution of burnout. In particular, the reciprocal effects of student perceptions of school climate on their engagement and burnout have been studied by Grazia (2022), who found that a favorable perception of school climate is a highly predictive factor of students’ engagement and a predictor of reduced levels of burnout. Molinari and Grazia (2021) conducted a similar study and found that perceptions of school climate varied among four student profiles, with the most peaceful and engaged students having the highest scores.

The relationship between growth mindset, academic grit, and academic burnout has also been explored, with Kim (2020) finding that academic burnout may be reduced as a result of a growth mindset, which is negatively mediated by academic grit. Novotny (2016) similarly investigated the role of mindset and grit in reducing counselor burnout and found that counselor self-efficacy acted as a buffer against burnout.

Yu et al. (2022) studied the moderating role of maladaptive emotion regulation strategies (ERS) in the relationship between motivation and burnout among EFL learners, finding that high levels of burnout were reported and that burnout showed an inverse correlation between motivation and maladaptive ERS. In a study on academic burnout in academic students with high and low levels of self-efficacy, Rahmati (2015) found an inverse correlation between self-efficacy, academic burnout, and its components. Maricutoiu and Sulea (2019) similarly explored the role of self-efficacy in the evolution of student engagement and burnout, finding that self-efficacy had a mediating impact on both. Gong et al. (2021) explored the relationship between resilience and student burnout in China and found an inverse correlation, with a decline in resilience resulting from a focus on the self and immature defense mechanisms against stress at both the individual and cultural levels.

The hypothesized model for this study proposes that perceived school climate and growth mindset are related to EFL student burnout among Chinese English language learners. It is hypothesized that a positive perceived school climate and growth mindset will also have a negative relationship with EFL student burnout.

The link between students’ growth mindset and their burnout is justified in light of the fact that growth mindset is related to resilience (Zeng et al., 2016; Calo et al., 2019), which is the ability to recover from stress and adversity. Students with a growth mindset believe that their abilities and intelligence can be developed, and they are more likely to view challenges as opportunities for growth rather than threats (Degol et al., 2018). This positive mindset may help students cope with academic and personal stressors, reducing their likelihood of experiencing burnout (Yeager and Dweck, 2012; Dweck et al., 2014). Also, growth mindset is associated with greater motivation and engagement in learning (Bedford, 2017; Hodge et al., 2018). Students with a growth mindset are more likely to embrace challenges and persist in the face of setbacks, which can lead to increased academic achievement and greater satisfaction with the learning process (Burnette et al., 2013). Additionally, growth mindset pertains to the development of adaptive coping strategies (Schroder et al., 2017). Students with a growth mindset are more likely to use problem-solving and emotion-focused coping strategies, which have been linked to better mental health outcomes (Yeager et al., 2019).

Also, the association between school climate and burnout can be justified in light of Self-Determination Theory (SDT, Deci and Ryan, 2008). Based on SDT, individuals’ psychological needs for autonomy, competence, and relatedness are essential for their optimal functioning and well-being. When students perceive their school climate as supportive, they feel more autonomous, competent, and connected to others in their learning environment. This sense of belonging and competence, in turn, can help protect students from experiencing burnout (Yang et al., 2020; Molinari and Grazia, 2021). Conversely, a negative school climate, characterized by lack of support, autonomy, and relatedness, may lead students to feel overwhelmed, demotivated, and disconnected, which can increase their likelihood of experiencing burnout.

Likewise, the relationship between students’ perceived school climate and their growth mindset can be theoretically justified through Social Cognitive Theory (SCT, Bandura, 1989). The SCT postulates that individuals learn by observing others and their environments, and that personal factors such as beliefs, values, and attitudes also influence learning and behavior. In this sense, students’ perceptions of the school climate, which includes the quality of the relationships and interactions among students and between students and teachers, can shape their beliefs about their abilities and potential for growth.

In light of the reviewed literature and the hypothesized model discussed above, the current study aims to investigate these relationships and provide insight into potential factors contributing to EFL student burnout. As such, this study is expected to make a significant contribution to the existing literature on student burnout and school climate, particularly in the context of EFL students. The findings of this study may also provide practical implications for educators and policymakers on how to improve school climate and promote student well-being in this particular context. Therefore, the following research questions are proposed:

1. What is the relationship between school climate and student burnout among EFL students?
2. What is the relationship between growth mindset and student burnout among EFL students?

How do school climate and growth mindset interact to influence student burnout among EFL students?

## 3. Methods

### 3.1. Participants

The current study recruited 412 intermediate adult English as a Foreign Language (EFL) learners from various language institutions in mainland China. The participants were selected using a convenience sampling method, where individuals who were easily accessible were chosen. The data collection was done through self-reported scales, which measured the affective factors that were the main focus of the study. The sample consisted of 153 male and 259 female students, with ages ranging from 18 to 25 years and a mean age of 22.08 (SD = 2.41). They reported that they have had from 8 to 13 years of English learning experience.

### 3.2. Instruments

As the participants of the study were at an intermediate level of English proficiency, it was deemed appropriate to administer the instruments in their original English versions, without translation. This decision was made to ensure that the intended meaning and nuances of the items were preserved and to avoid potential issues with the accuracy and validity of the translations.

#### 3.2.1. Foreign language burnout

In this study, the research used a survey instrument designed to assess burnout in English as a foreign language (EFL) students, known as the “Maslach Burnout Inventory-EFL Student Survey.” This survey was adapted from the “Maslach Burnout Inventory-Student Survey (MBI-SS)” (Schaufeli et al., 2002) to better suit the context of EFL learning. The modified survey consisted of 10 items and three dimensions: Exhaustion (Ex, four items), Cynicism (Cy, three items), and Reduced Efficacy (RE, three items). Participants responded to the items on a 7-point Likert scale that ranged from “completely disagree” to “completely agree.” A representative item from the survey is “I have become less enthusiastic about my English studies.” The overall reliability of the survey was found to be high, with an alpha coefficient of 0.92.

### 3.3. Classroom climate

The classroom climate perception of the participants was measured using a tool developed by Joe et al. (2017). The scale consists of three dimensions, which have been validated by Joe et al. (2017) as having high reliability coefficients: Teacher’s Academic Assistance (TAS, with 3 elements, reliability coefficient of *α* = 0.84), Teacher’s Emotional Support (TES, with 4 elements, reliability coefficient of *α* = 0.84), and Classroom’s Mutual Respect (CMR, with 2 elements, reliability coefficient of *α* = 0.71). Participants were asked to rate each element on a 5-point Likert scale, where 1 represented “strongly disagree” and 5 represented “strongly agree.”

#### 3.3.1. Growth mindset scale

The participants’ growth mindset was assessed using a measure developed by Papi et al. (2019). This measure was based on Dweck et al. (2014) original mindset scale and was modified to fit the context of language learning. It consisted of four items rated on a 6-point Likert scale, ranging from 1 (strongly disagree) to 6 (strongly agree). One example item from the scale was “I can significantly improve my language learning abilities.” The reliability of the scale, as measured by using Cronbach’s alpha formula, was found to be 0.89 (as presented in Table 1).

**Table 1:** Measurement model fit indices.

	χ^2^	Df	χ^2^/df	CFI	TLI	RMSEA	α
School climate	109.65	55	1.99	0.93	0.92	0.06	0.81
Growth mindset	78.64	42	1.87	0.97	0.96	0.03	0.89
Burnout	257.24	133	1.93	0.95	0.94	0.04	0.92

χ^2^, chi-square; Df, degrees of freedom; CFI, comparative fit index; TLI, tucker-lewis index; RMSEA, root mean square error of approximation; α, coefficient alpha.

### 3.4. Procedure

The present study was a non-experimental research, in which the researcher developed electronic versions of three questionnaires to assess participants’ growth mindset, perceptions of school climate, and experiences of student burnout. Data was gathered through a Chinese online survey platform, and the survey link was disseminated to English language learners across various regions in mainland China. The online survey was designed with clear instructions and provided information on how to complete the questionnaire. Participants were requested to participate voluntarily and grant informed consent, with the assurance of the confidentiality of their personal information (Table 2).

**Table 2:** Descriptive statistics.

	*M* (SD)	1	2	3
(1) School climate	4.36 (0.92)	1.00		
(2) Growth mindset	4.25 (1.16)	0.24*	1.00	
(3) Burnout	3.03 (0.99)	−0.44**	−0.34**	1.00

The significance levels for the correlations are denoted as follows: *p < 0.05, **p < 0.01.

## 4. Data analysis and results

In the present study, the data was analyzed using Statistical Package for the Social Sciences (SPSS 24) and Analysis of Moment Structures (AMOS 23) software (Kline, 2015). Confirmatory Factor Analyses (CFAs) and Structural Equation Modeling (SEM) were employed to assess the validity of the latent constructs and to test the hypothesized structural theories. Data screening was conducted to investigate the presence of missing values, outliers, and normality in the data (Kline, 2015). The missing values were addressed through the expectation–maximization algorithm, which filled in the missing data with random values (Kline, 2015). Outliers were determined through standard scores and Mahalanobis $D^{2}$ values and values that were non-normal (kurtosis and skewness outside the range of −1 to +1) were removed (Kline, 2015), resulting in 403 valid cases for further analysis. Also, Preliminary analyses were conducted to examine the associations between the demographic variables (i.e., gender, age, and years of learning English as a foreign language) and the study variables of interest. It was found that no significant correlations were observed between these demographic variables and the study constructs. Therefore, these variables were not included as covariates in the main analyses.

The validity of the measurement models was assessed by conducting CFAs and evaluating the fit indices, such as Chi-square divided by degree of freedom (*χ*^2^/df), Comparative Fit Index (CFI), Tucker-Lewis Index (TLI), and Root Mean Square Error of Approximation (RMSEA) (Hu and Bentler, 1998; Kline, 2015). Adequacy was considered when *χ*^2^/df was less than 3, CFI and TLI were greater than 0.90, and RMSEA was less than 0.08 (Hu and Bentler, 1998). In cases where the measurement models failed to meet these standards, items with low loadings from the questionnaires were eliminated (one items from the school climate measure and one items- from the burnout measure). The modified models showed good fit and reliability, as demonstrated by coefficient alphas exceeding 0.70 (Kline, 2015). Subsequently, descriptive statistics and correlation coefficients were calculated for the constructs.

### 4.1. SEM analysis

**Figure 1:**
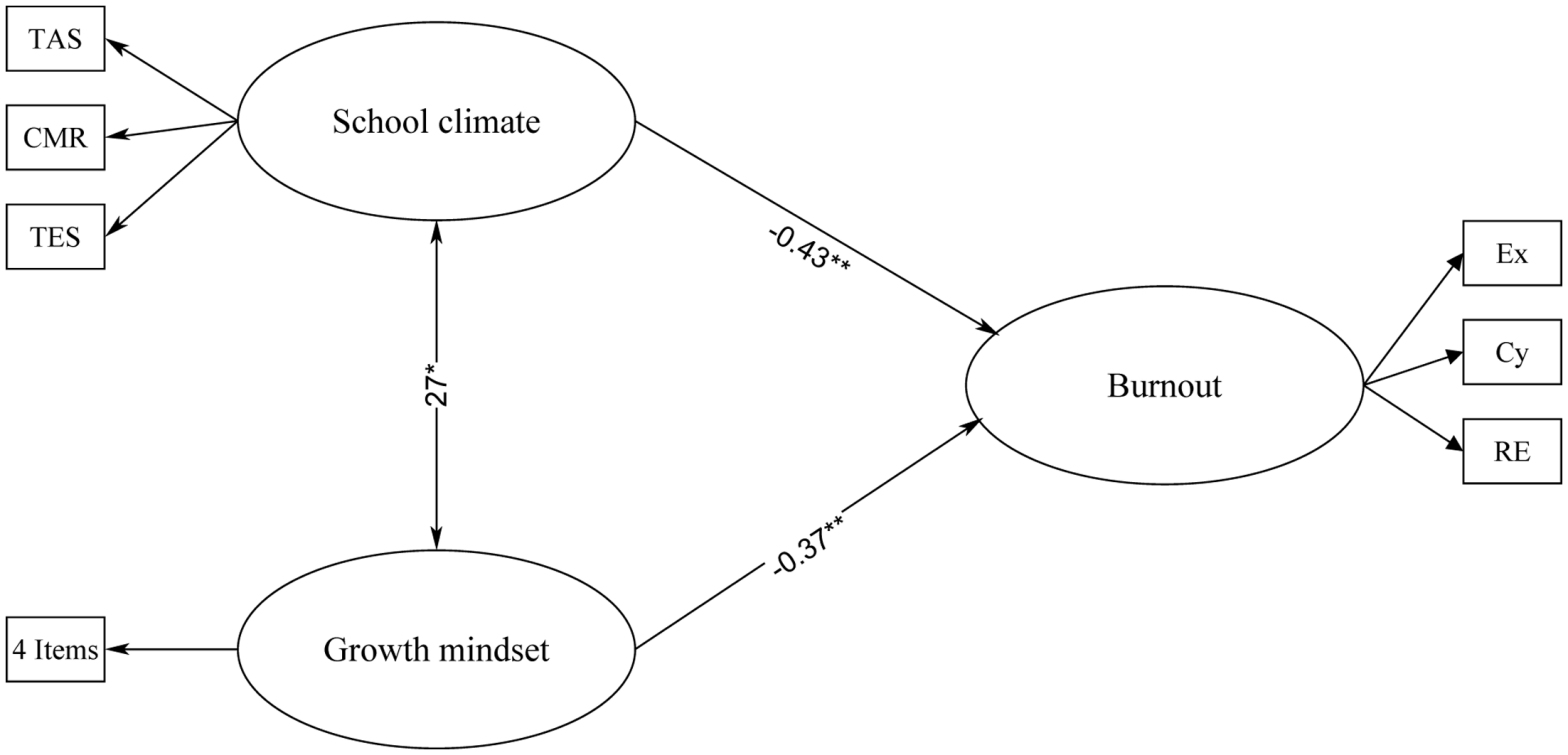
The final model of EFL student Burnout. *p < 0.05; ***p < 0.001.

In this study, the proposed structural model was analyzed through the utilization of maximum likelihood estimation and variance–covariance matrices as inputs, using the AMOS 23 software. The results demonstrated the significance of the path coefficients, with a value of p less than 0.05, and the appropriateness of the fit indices. The SEM analysis confirmed all the hypotheses of the proposed model, as depicted in Figure 1. To provide additional insight into the results, the effect size (ES) of the latent constructs was calculated using Cohen’s f2 measure.

The model fit indices for the SEM analysis were *χ*^2^ = 243.56, df = 87, *χ*^2^/df = 2.8, RMSEA = 0.07, CFI = 0.95, and TLI = 0.93, indicating a reasonable and acceptable fit of the model to the data. These indices provide strong evidence for the reliability of the SEM analysis and support the interpretation of the results regarding the relationships between student growth mindset, school climate, student engagement, and EFL student burnout (Kline, 2015).

The results of the SEM analysis, as depicted in Figure 1, revealed a significant relationship between student growth mindset and English as a Foreign Language (EFL) student burnout (*β* = −0.37, *R^2^* = 0.13, f2 = 0.15, average ES). Furthermore, the school climate was identified as a stronger predictor of student engagement (*β* = −0.43, *R^2^* = 0.18, *f2* = 0.21, large ES). Additionally, the results demonstrated a significant association between school climate and growth mindset (*β* = 0.27, *R^2^* = 0.07).

## 5. Discussion

The objective of the present study was to examine the impact of school climate and growth mindset on student burnout among Chinese EFL students. The findings of the study are noteworthy, particularly with regard to the relationship between school climate and student burnout. The results showed that there was a significant association between school climate and student burnout. This result is consistent with previous research (Fatou and Kubiszewski, 2018; Yang et al., 2020; Molinari and Grazia, 2021; Grazia, 2022) that found that students’ engagement had a positive association with their school climate perceptions, while student burnout had a negative association with their perceptions of school climate. For example, Molinari and Grazia (2021) found that students with the “peacefully engaged” profile, as the most adaptive pattern of engagement and burnout, did not display symptoms of burnout in their schoolwork and felt energetic about participating in school activities. The results of this study provide support for the theoretical framework of SCT (Bandura, 1989) as a way to understand the relationship between students’ perceived school climate and their growth mindset. SCT proposes that personal factors (e.g., cognitive and affective processes) and environmental factors (e.g., social and physical contexts) interact with one another in a reciprocal and dynamic way, which can shape an individual’s behavior and beliefs. In this case, the school climate, as a key environmental factor, can influence students’ cognitive processes, such as their beliefs about the nature of intelligence and learning, which are central to the concept of growth mindset.

The findings of the present study are in line with those of Salmela-Aro et al. (2008), who found that a negative school climate was positively associated with school burnout while school support and positive motivation from teachers were negatively associated with school burnout. The significant impact of school climate on student burnout may be attributed to the role of learning environments in influencing students’ engagement in the learning process. Under a multi-level multi-informant structural model, students’ engagement may be enhanced by a positive school climate, which supports the idea that students are more likely to be engaged in schools that provide higher levels of structure and support, resulting in higher academic success. This is consistent with some research studies (Geller et al., 2013; Cornell et al., 2016) that suggest that a positive school climate can lead to increased self-concept and self-regulation, which in turn improves students’ study engagement and academic achievement.

The relationship between school climate and student engagement has been established by previous literature (Wang and Degol, 2016; Fatou and Kubiszewski, 2018; Del Toro and Wang, 2021). Additionally, Wang and Holcombe (2010) stated that a positive representation of school climate has a positive impact on students’ outcomes and behavioral and emotional engagement, leading to increased participation in school activities, a greater sense of school belonging, and increased appreciation of school. Furthermore, a positive school climate positively affects students’ engagement and academic achievement (Lombardi et al., 2019), which is consistent with previous research (Pong and Zeiser, 2012; Bear et al., 2018a,b; Storlie and Toomey, 2019). Conversely, a negative school climate leads to increased student disengagement or burnout in the educational system (Grayson and Alvarez, 2008).

The findings of the present study support previous research (Fredricks et al., 2004; Wang and Holcombe, 2010; Pilkauskaite-Valickiene et al., 2011; Wang and Eccles, 2012; Shih, 2015, among others) that revealed that students’ academic coping strategies are influenced by both classroom structure and peer support, and their burnout experiences are in turn influenced by the way they cope with academic stress. Provision of structure and peer support reduces feelings of inadequacy and enhances success in school activities, leading to lower levels of emotional exhaustion and cynicism, two aspects of burnout.

The findings of this study indicate that a growth mindset has a substantial interconnection with student burnout. As previous research suggests (Yeager and Dweck, 2012; Dweck et al., 2014; Zeng et al., 2016; Sanguras, 2017; Kim, 2020), students who adopt a growth mindset experience less academic burnout and display higher levels of self-confidence and resilience in the face of boredom (Wang, 2023) and exhaustion, leading to improved academic performance and achievement. This conclusion is supported by prior studies (Burnette et al., 2013; Hodge et al., 2018; Zhao et al., 2018; Tang et al., 2019), which highlight that exhibiting a growth mindset, such as by having a more positive outlook on performance and making consistent efforts, can help prevent academic burnout. The association between school climate and burnout can be theoretically explained by SDT (Deci and Ryan, 2008). According to SDT, individuals have three basic psychological needs that must be fulfilled for optimal functioning: autonomy, competence, and relatedness. A positive school climate can provide opportunities for students to experience autonomy by allowing them to have a sense of control over their learning and decision-making. This, in turn, can increase their sense of competence and relatedness, as they feel supported and connected to their peers and teachers. On the other hand, a negative school climate can limit students’ opportunities for autonomy, competence, and relatedness, which can lead to feelings of burnout. Additionally, the findings are partially consistent with previous studies (Klussman et al., 2020) that show that stress mindset and self-connection predict different types of student burnout. Specifically, both personal and school-related burnout results from a stress mindset where stress is perceived as debilitating.

The study also found that the school climate has a small effect on students’ growth mindsets. This is in line with previous research (Luo et al., 2019; Yeager et al., 2019; Mesler et al., 2021; Yu et al., 2022), which shows that the school and classroom settings contribute to 5–8.7% of the variance in students’ growth mindset. This finding also aligns with SCT (Bandura,1989), which postulates that individuals’ beliefs and attitudes are influenced by their social environment. Specifically, according to SCT, individuals learn from observing the behavior and attitudes of others in their environment, including teachers and peers. In the context of the current study, students may be influenced by the attitudes and beliefs of their teachers and peers regarding the importance of effort, learning from mistakes, and the value of challenges, which are key components of a growth mindset. However, this finding contradicts previous literature (Thomas et al., 2019), which suggests that there is a significant difference in institutional and personal growth mindset between public and private school students. Lastly, this conclusion is in contrast to prior studies (Dalbert and Stoeber, 2005; Peter and Dalbert, 2010) that suggest a strong correlation between belief in a just world and school climate, as students with a belief in a just world tend to feel a stronger attachment to their school and view it as a place where effort leads to achievement.

The present research sought to examine the impact of school climate and growth mindset on student burnout among Chinese EFL students. The study might make several important contributions to the existing literature. Firstly, it boldfaces the significant effect of school climate on student burnout, consistent with previous research. The findings add to this body of research by showing that a positive school climate can enhance students’ engagement in the learning process, resulting in higher academic success. It also demonstrates that a growth mindset has a substantial impact on student burnout, suggesting that adopting a growth mindset can lead to improved academic performance and achievement, as well as higher levels of self-confidence and resilience in the face of boredom and exhaustion.

Regarding the specific advances of this work, the present outcomes offer new insights into the contribution of school climate and growth mindset to student burnout among EFL students. These findings might also contribute to the ongoing discussion on the relationship between school climate and student engagement, highlighting the importance of providing higher levels of structure and support to enhance student engagement and academic success. Additionally, the results shed light on the impact of growth mindset on academic burnout, adding to the growing body of research on this topic.

Also, the findings provide new insights into the specific context of EFL students in China. It seems necessary to consider the unique challenges faced by EFL students, such as language barriers and cultural differences, when examining the relationship between school climate, growth mindset, and student burnout. By focusing on this specific population, this research offers practical implications for educators and policymakers aiming to enhance student well-being and academic success.

## 6. Conclusion

The purpose of this study was to examine the relationship between student burnout, school climate, and growth mindset among Chinese EFL students. The results of the study indicated that student burnout was significantly predicted by both school climate and growth mindset. School climate, as a crucial factor in creating a positive learning environment that affects physical and mental well-being, academic engagement, and achievement, had a significant impact on student burnout. Meanwhile, growth mindset, as a protective factor that enables students to overcome challenges and setbacks, also significantly influenced student burnout. However, it was found that school climate had a minimal effect on growth mindset.

Theoretically, the study contributes to the literature on student burnout and provides evidence for the relationship between student burnout and two important factors, perceived school climate and growth mindset, in the context of EFL learning among Chinese students. This adds to the understanding of how these factors may contribute to student burnout and provides a foundation for future research in this area. Practically, the results of this study have important implications for EFL teachers, school administrators, and policymakers. The findings suggest that promoting a positive school climate and fostering student growth mindset can help reduce student burnout in EFL settings. This means that schools and teachers can take practical steps to reduce student burnout by creating a supportive and positive learning environment and encouraging students to adopt a growth mindset. To achieve this, schools and teachers can implement practical initiatives, such as conducting regular surveys to assess students’ perceptions of the school climate and addressing areas of concern identified by students. Additionally, teachers can integrate growth mindset practices into their lesson plans by providing students with opportunities to set goals, receive feedback, and reflect on their learning progress. School administrators and policymakers can also support these efforts by providing resources, such as professional development opportunities and funding for school-wide initiatives, that promote a positive school climate and growth mindset. Given the multicultural nature of schools and colleges, these findings emphasize the significance of this research for EFL teachers, college administrators, and others involved in the educational system. These individuals are responsible for creating a supportive and inclusive school environment by offering courses that focus on diversity and equity. They can also help shape students’ beliefs about learning by providing training sessions, such as hands-on activities and discussions, that foster growth mindset and academic achievement. The results of the study provide useful recommendations for teacher educators, policymakers, and other stakeholders to promote a positive learning environment by implementing comprehensive programs and practical initiatives, such as those mentioned above. This will improve students’ academic performance and engagement and help prevent burnout among EFL students.

Nevertheless, there are also some limitations to this study that should be taken into consideration. Although the study examined the distal factors of perceived school climate and growth mindset as possible factors contributing to student burnout, it would be beneficial to include more proximal variables, such as motivation and emotions, to gain a more comprehensive understanding of this phenomenon. Moreover, although the Maslach model (Maslach et al., 1997) is widely used to measure burnout, it may not capture all aspects of this phenomenon (Shirom, 2005). Furthermore, it is acknowledged that the use of multidimensional scales can provide a better understanding of complex constructs like classroom climate, and it is recommended that future researchers conduct such studies. Likewise, this study did not examine the potential links between the sub-dimensions of school climate and burnout. It is possible that certain aspects of the school climate, such as teacher-student relationships or academic demands, may be more strongly related to burnout than others. Future research could explore these sub-dimensions to gain a more nuanced understanding of the association between school climate and burnout.

Additionally, the sample used in the study consisted of intermediate English language learners from China, and the findings may not generalize to other populations or settings. Also, the study was based on self-reported data, and as such, may be subject to response bias and social desirability effects. Finally, this study was cross-sectional in nature, and as such, causality cannot be established. Further longitudinal and experimental studies are needed to better understand the complex relationships between these factors and student burnout.

In conclusion, this research highlights the negative relationship between perceived school climate, growth mindset, and student burnout among EFL students in China. These outcomes have important implications for EFL teachers, school administrators, policymakers, and other stakeholders involved in the educational system. By promoting a positive school climate and fostering a student growth mindset, practical steps can be taken to reduce student burnout and improve academic performance and engagement. This research also contributes to the literature on student burnout and provides recommendations for comprehensive programs and practical initiatives to create a supportive and inclusive learning environment, with potential for future research in this area.

## Data availability statement

The original contributions presented in the study are included in the article/supplementary material, further inquiries can be directed to the corresponding author.

## Ethics statement

Ethical review and approval was not required for the study on human participants in accordance with the local legislation and institutional requirements. Written informed consent from the patients/participants or patients/participants legal guardian/next of kin was not required to participate in this study in accordance with the national legislation and the institutional requirements.

## Author contributions

The author confirms being the sole contributor of this work and has approved it for publication.

## Funding

This work was supported by a study on the Professional Development System of College Foreign Language Teachers in the Era of Intelligent Education.

## Conflict of interest

The author declares that the research was conducted in the absence of any commercial or financial relationships that could be construed as a potential conflict of interest.

## Publisher’s note

All claims expressed in this article are solely those of the authors and do not necessarily represent those of their affiliated organizations, or those of the publisher, the editors and the reviewers. Any product that may be evaluated in this article, or claim that may be made by its manufacturer, is not guaranteed or endorsed by the publisher.
